# Factors influencing female students’ entrepreneurial intention in vocational colleges: A multi-group analysis based on household income

**DOI:** 10.1371/journal.pone.0304232

**Published:** 2024-05-23

**Authors:** Rong Wang, Rubing Liu

**Affiliations:** School of Architecture and Engineering, Taizhou Polytechnic College, Taizhou, Jiangsu, China; Babes-Bolyai University, Cluj-Napoca, ROMANIA

## Abstract

Female entrepreneurs have irreplaceable status and essential significance in entrepreneurship research. Improving females’ entrepreneurial intentions is an important topic in this area. Accordingly, this study, based on the theory of planned behavior, investigates the factors that affect female students’ entrepreneurial intention at China’s vocational colleges and whether household income moderates the relationship between entrepreneurial education, attitude, competence, self-efficacy and entrepreneurial intention. 2149 females from vocational colleges in Guangdong Province, Zhejiang Province, and Jiangxi Province were randomly chosen to participate in the study. They had taken part in entrepreneurial courses throughout 2021–2022. In addition, data were analyzed by structural equation modeling partial least squares. The results demonstrate that entrepreneurial education did not directly affect female students’ intentions. Entrepreneurial competence, self-efficacy, and attitude positively affect entrepreneurial intention. It is further concluded that household income significantly moderates the relationship between entrepreneurial education, attitude, competence, and intention. However, there is no significant difference in the relationship between self-efficacy and entrepreneurial intention between high and low-household-income students. While females continue to confront sexism in the workplace, it is crucial that we conduct empirical research into the factors influencing female entrepreneurial intention to boost economic growth and gender parity. This research helps bridge a gap in the prior literature and adds substantial value to encouraging female entrepreneurs.

## 1. Introduction

The ongoing pandemic and a surge in college graduates have exacerbated the employment crisis domestically. To mitigate this employment strain, entrepreneurship, characterized by its low entry barriers and adaptability, emerges as a viable solution, potentially spurring economic revival [1]. Significantly, entrepreneurship is increasingly recognized as a key driver of global economic expansion [2]. Consequently, there has been a surge in scholarly research focusing on entrepreneurial activities among various demographics, including returning migrant workers [3], researchers [4], and students [3,4]. Notably, students are considered central to entrepreneurial endeavors, attributed to their inherent innovative and adventurous qualities. Moreover, as highlighted in the Outline for Women’s Development in China (2021–2030), vocational college students are increasingly becoming the backbone of student entrepreneurship. This trend is likely influenced by vocational colleges’ emphasis on practical skills, hands-on experience, and industry connections, offering them a competitive edge in entrepreneurial ventures [5,6]. Within the realm of social psychology, intentions are widely acknowledged as reliable predictors of behavior. Therefore, examining the entrepreneurial intentions (EI) of vocational college students is essential, as it sheds light on the underlying motivations and psychological mechanisms driving such intentions.

The statistical report of the Outline for Women’s Development in China (2021–2030) reveals that female students comprise 50.2% and 57.7% of the population in vocational and adult education institutions, respectively. This majority presence of female students in higher education underscores their growing interest in advanced studies and career advancement, reflecting societal progress in gender equality and female empowerment. Consequently, it becomes crucial to examine the EI of women in vocational colleges [7]. This examination is vital for several reasons. Firstly, fostering entrepreneurship among female vocational college students challenges traditional career norms and roles, addressing issues like gender stereotypes, discrimination, and the underrepresentation of female role models [8]. Secondly, female entrepreneurship plays a significant role in driving economic growth [9]. Analyzing the EI of female students in these institutions provides valuable insights into their entrepreneurial drive and creative potential across various industries, thereby catalyzing new opportunities for sustainable socio-economic development [10].

The pivotal role of female entrepreneurs in economic growth has catalyzed extensive research on the EI of female students, as evidenced by works from Khan [11]. These studies predominantly explore the factors influencing women’s EI from two angles: internal and external factors. Regarding internal factors, Zhou argue that individual cognitive attributes like self-efficacy significantly influence the EI of Italian female students [12]. Dong highlight the importance of personal traits, such as alertness and risk-taking abilities, in shaping the EI of female engineering students [13]. Additionally, Margaça underscore the influence of personal background elements, including work experience, marital status, and intrinsic motivation, on EI [14]. From an external perspective, research by Lin and Dong indicates that university environments and support systems, encompassing aspects like entrepreneurial training and context, critically affect students’ attitudes and perceived behavioral control, thereby impacting their EI [13,15]. Li emphasize the significance of financial resources and entrepreneurship education in influencing EI [16]. Furthermore, several scholars have expanded the EI model to integrate additional moderating variables, including social support and entrepreneurship education, further enriching our understanding of the factors that shape EI [4,16].

Although there has been increased interest in researching female entrepreneurs, current limitations in the field still hinder the advancement of EI studies [15,16]. A thorough examination of existing literature reveals an often-overlooked aspect: the influence of funding on the EI of women. The Outline for Women’s Development in China (2021–2030) highlights that college student entrepreneurs regard funding as a crucial factor for entrepreneurial success, assigning it a significant weightage of 46.2% in a survey [17]. This underscores that funding scarcity poses a considerable barrier to college students’ entrepreneurial endeavors. This challenge can be attributed to the initial costs associated with entrepreneurship, such as renting office space, hiring personnel, and sustaining working capital, all of which are exacerbated by the absence of a stable income and savings [18]. Additionally, the report indicates that only a small fraction (10.6%) of funding for young entrepreneurs in China is sourced from bank loans or financial institutions, while a substantial majority (75.0%) originates from household savings. This reality positions household income as the primary funding source for student startups. Given these insights, this study proposes to include household income as a moderating factor in the examination of its impact on the EI of female students in vocational colleges. This approach aims to provide a deeper understanding of how household income influence the EI and capabilities of these students.

Existing research suggests that in entrepreneurial education, it is particularly important to clearly distinguish an individual’s intrinsic confidence, or entrepreneurial self-efficacy, for understanding and predicting entrepreneurial behavior [19–21]. Therefore, this study employs the Theory of Planned Behavior as the foundational framework, with necessary expansions and adjustments made, particularly by considering entrepreneurial self-efficacy as an independent variable. This research seeks to develop a hypothesis model of EI among female college students in higher vocational institutions. Meanwhile, three research questions are put forth: (1) What factors influence the EI of female vocational college students? (2) To what extent do these factors account for the variance of the EI of female students? (3) Does household income significantly moderate between various independent variables and EI?

Moreover, this study is structured as follows to explore the issues mentioned above: Firstly, the conceptual model constructed for the research is presented in Section two, along with the hypotheses. Secondly, the methods are detailed in Section three of the study. Meanwhile, Section four interprets the outcomes of the data analysis. Finally, conclusions, discussion, and implications for further research are proposed.

## 2. Literature review and hypothesis

### 2.1 Literature review

#### 2.1.1 The theory of planned behavior (TPB)

TPB, originating from the Theory of Reasoned Action in 1980, aims to predict an individual’s intention to engage in a specific behavior at a particular time and place [22]. This theory encompasses five variables: attitude, subjective norms, perceived behavioral control, behavioral intention, and behavior [22]. Here, attitude refers to an individual’s favorable or unfavorable evaluation of the behavior in question; subjective norms pertain to beliefs about whether most people approve or disapprove of the behavior; perceived behavioral control denotes an individual’s perception of the ease or difficulty of performing the behavior of interest; behavioral intention represents the motivational factors that influence a specific behavior, with stronger intentions increasing the likelihood of performing the behavior.

In recent years, TPB has been applied in the entrepreneurial field to predict the EI of university students [23–25], vocational college students [26], high school students [27], disabled individuals [28], and researchers [29]. Additionally, scholars have attempted to integrate the TPB with other theoretical frameworks/models, such as self-efficacy theory [30], self-determination theory [31], the Triple Helix Model [29], the organismic theory of motivation [31], and social cognitive theory [23], to develop new theoretical frameworks aimed at a deeper explanation of the factors influencing EI. Moreover, existing studies have utilized various research tools to explore the factors impacting individual entrepreneurial intentions, such as structural equation modeling (AMOS, SMART PLS, LISREL) [23,24,30], meta analysis—structural equation modeling [32,33], citespace [34], and fuzzy-set qualitative comparative analysis [28]. Finally, beyond attitude, perceived behavioral control, and subjective norms, studies have shown that factors influencing individual EI also include creativity [25], social emotional competence [23], entrepreneurial self-efficacy (ESE) [23], student internship motivation [24], perceived entrepreneurial capacity [27], entrepreneurial orientation [30], opportunity recognition [30], perceived feasibility [35], entrepreneurial knowledge [32], government support [29], and industrial and financial support [29]. A comprehensive review of the literature has shown that while previous studies primarily focused on predicting factors influencing entrepreneurial intentions among university students, they did not specifically address the entrepreneurial intentions of female students in vocational colleges. Additionally, existing research did not cover the moderating role of household income between predictive factors and entrepreneurial intentions.

#### 2.1.2. EE

Entrepreneurial education can be defined as “intentional intervention by educators in the lives of learners, to impart entrepreneurial qualities and skills, ensuring that learners can survive in the business world” [36]. This definition indicates that entrepreneurial education involves the use of, creation, and implementation of innovative and proactive approaches within a learning environment. A successful entrepreneur is not due to his innate factors but requires knowledge, skills, and specific practical experience [4]. Therefore, entrepreneurship needs more nurturing. EE was one method for reducing the gender disparity in entrepreneurship [37,38]. It aimed to prepare students to start businesses by teaching entrepreneurship-related courses and practical activities, thereby increasing the likelihood of entrepreneurship success [39]. It was the primary source of entrepreneurial knowledge and skills [40]. Increasing concern regarding EE has triggered a discussion regarding whether EE can affect EI. On the one hand, some researchers have argued that EE enhances the EI of college students [41,42]. Li argued that students would exhibit more EI if they received EE perceived as high quality and engaging [16]. It has been demonstrated that the better the EE offered by colleges, the greater the number of student entrepreneurs it inspires, possibly because it differs from general education [4]. It focuses on fostering student creativity for innovative problem-solving and self-employment skills. Duong investigation reached a similar conclusion [43]. However, other researchers argue that EE for college students has no significant effect on EI [44]. Kusumojanto explored the factors influencing entrepreneurial intentions of vocational students and showed that entrepreneurship education for vocational students did not significantly predict entrepreneurial intentions [44].

In addition, these researchers contended that gender moderates the association between EE and EI [16]. Males are more inclined to use available resources to engage in EI than females. In his research, Cao strongly emphasized the value of EE for females [45]. In addition to helping them obtain a degree, it can allow them to acquire problem-solving skills that will be useful in their future entrepreneurial careers. Lastly, an empirical study conducted by Duan also concluded that EE in colleges had been shown to have a significant and positive effect on the EI of students, and the knowledge of entrepreneurship students obtain in colleges was more conducive to the application of entrepreneurial practice [46].

#### 2.1.3 EC

The concept of EC was first articulated by Chandler and Hanks [47]. It was described as “the competence of entrepreneurs to discover, anticipate, and seize opportunities, which was a practical ability.” Other scholars also believed that entrepreneurship was a process of continuous development and that entrepreneurs must continually acquire resources to adapt to this process of rapid growth; this ability to receive resources was initially categorized as EC [48,49].

In contrast to their male counterparts, the femininity of female entrepreneurs gives them the ability to handle related matters more softly and gives them the interpersonal skills necessary to form cordial, productive partnerships [50]. Empirical research by Mustafa and Treanor [50] demonstrated that female entrepreneurs often ranked their interpersonal and social abilities as among their greatest strengths but had less power in financial skills. Other researchers also showed similar results [10,16]. Although there were few in-depth studies on the EC of females, there were numerous studies on EC in general. There was sufficient theoretical support to demonstrate that EC was the primary factor influencing EI and determining the success of entrepreneurship [51–53]. It was not only the embodiment of the comprehensive quality of entrepreneurs but also determined EI by influencing the expectation of success. As contended by **Chu**, individual EC was one of the critical factors affecting entrepreneurial decision-making and EI [23]. **Chien-Chi** are convinced that enhancing EC can lower the risks associated with entrepreneurship, raise their interest in engaging in it, and increase their chances of success [54]. At the same time, from an alternative viewpoint, some studies believed that individuals with strong EC had stronger risk perception abilities but were more reluctant to start a business because entrepreneurship is a high-risk activity [26,55,56].

### 2.2 Hypothesis

Entrepreneurial attitude (ATE) refers to a person’s view and assessment of entrepreneurial outcomes, which can shift depending on people’s appraisals of the positive outcomes of EI [57]. A study by Jena determined that EE affects ATE [39]. The primary objective of EE is to alter students’ perceptions about entrepreneurship to increase their understanding of it. Tomy Pardede contended that ATE is affected not only by previous experience but also by EE [58]. The findings of Liu strengthen the opinion of those who believe that EE can increase students’ knowledge [37], enhance their self-confidence, and improve their awareness that “entrepreneurship is a feasible option.” Based on the above discussion, the following hypothesis is proposed:

**H1:** The EE and ATE of female vocational college students are positively related.

ESE refers to a person’s confidence to successfully carry out specific entrepreneurship-related duties [59]. Several empirical studies have investigated the relationship between EE and ESE [60,61]. According to the study of Soomro and Shah, in addition to objectively enhancing entrepreneurial skills and providing practical opportunities, EE also subjectively strengthens students’ perception and control over entrepreneurial activities [62]. Another study concluded that those who have undergone EE typically have substantially higher levels of self-efficacy in EE than those who have not [61]. Additionally, those who complete the EE task have a high level of ESE because they are confident in their upcoming EI. As a result, it is supposed that:

**H2:** The EE and ESE of female vocational college students are positively related.

Empirical studies have also revealed that a direct relationship exists between EC and ESE [10,63]. For instance, Chien-Chi stated that ESE is highly affected by EC, according to the survey of EI of college students participating in social entrepreneurship [54]. His study found that individuals with greater EC are more likely to possess business acumen and entrepreneurial thinking, thus creating more competitive products and achieving optimal benefits. This increases confidence in their EI, boosting their sense of ESE. Furthermore, Svotwa investigated the EI of African youth. The results indicate that EC has been demonstrated in other research to impact the ESE directly [63]. It makes sense that EC can affect ESE. Thus, in the studied setting, we postulate:

**H3:** The EC and ESE of female vocational college students are positively related.

According to empirical research, research has shown that EC and ATE are directly related [62,64]. For instance, San-Martín in his research reported that EC had a direct effect on individuals’ ATE [65]. Similarly, EC was incorporated into the TPB as a separate element and exerted a significant positive impact on ATE in the research conducted by Wardana [64]. According to Ferreira [66], the empirical literature on the relationship between EC and ATE was severely lacking, especially among students at vocational colleges. Consequently, the following hypothesis is proposed:

**H4:** The EC and ATE of female vocational college students are positively related.

Individuals with a solid ATE can better succeed in the business world [39,64,67]. Yang demonstrated through the results of a structural test that individuals with greater independence and greater risk tolerance will have a stronger ATE and are more likely to start a business [67]. However, individuals who frequently fear taking risks and avoid work responsibilities tend to work in more stable environments. Analogous research outcomes were reported by Krieger, who found a relationship between ATE and EI [68]. According to the research above findings, the study will predict the following hypothesis:

**H5:** The ATE and EI of female vocational college students are positively correlated.

Sufficient empirical evidence suggests that ESE may boost EI [16,19,69,70]. ESE was considered a crucial cognitive element connecting environmental conditions with EI [71]. Cai et al. concluded that high levels of ESE lead people to aggressively seek out and seize opportunities, which can stimulate EI [72]. Li pointed out that an individual’s level of ESE is the primary determinant of whether they will become an entrepreneur [16]. In addition, empirical studies by Shaheen and AL-Haddad argued that individuals with a high sense of ESE are more optimistic about the viability of enterprises and have a more positive attitude towards achieving entrepreneurial goals, which can promote EI [70]. Therefore, this study hypothesizes:

**H6:** The ESE and EI of female vocational college students are positively related.

Many prior studies have found that EE positively correlates with EI [16,40,72]. considered that the improvement of ability cannot be separated from the promotion of EE, which can help individuals improve their entrepreneurial skills and compensate for the disadvantages caused by lack of experience as much as possible. According to research on the impact of environmental factors on EI, compared with individuals who have not received EE, participants in EE can access a large number of entrepreneurial information and have a greater impact on entrepreneurial outcome expectations and EI. Researchers like Wardana also proposed that EE is the best predictor of EI [64]. Hence, the following hypothesis is proposed:

**H7:** The EE and EI of female vocational college students are positively related.

There is a positive correlation between EC and EI [73] (Zhang & Zhu, 2018). Silveyra et al. point out that entrepreneurs may encounter and overcome challenges during their actual entrepreneurial activity, necessitating exceptional EC [73]. It was concluded by Behling and Lenzi that there was a positive relationship between an individual’s level of EC and their propensity to engage in EI [74]. In addition, Reis believe that successful entrepreneurs need to have the ability to foresee and deal with future challenges in their businesses [52]. Based on the discussion above, this research concludes that people with more diverse and well-rounded EC are more likely to succeed in entrepreneurial practice. Therefore, the following hypothesis is proposed:

**H8:** The EC and EI of female vocational college students are positively related.

They are drawing on the existing research conclusions and the above analysis. As the primary source of students’ starting funds for entrepreneurship, household income may be the moderator of the relationship between EC and EI [66,75]. So, for students with more EC, a more considerable household income will assist them in satisfying their needs for start-up funds, increasing their entrepreneurial success rate [76]. On the contrary, higher household income for entrepreneurs with low EC can not only meet the shortage of start-up funds but also improve the possibility of EI by increasing their chances of trial and error and anti-risk ability [77]. Therefore, the following hypothesis is proposed:

**H9a:** Household income moderates the impact of EC on EI.

Entrepreneurs with adequate financial backing and a high self-efficacy are highly motivated to engage in EI. Entrepreneurs with high funds often have high household incomes and family assets [78]. Their families can provide a lot of financial support for enterprises, such as raising the registered capital of enterprises and hiring professionals [76]. The resource advantage brought by this high economic level will further stimulate entrepreneurs with high self-efficacy, so the financial support given by household income is the catalyst for achieving entrepreneurial success [79,80]. As a result, the following hypotheses arise:

**H9b:** Household income moderates the impact of ESE on EI.

Family is a significant source of star-tup funds for emerging and young entrepreneurs [77]. Females from high-income families are more likely to have access to examples of successful entrepreneurs and get more help from social resources. This boosts their confidence and motivation to start a business [37]. However, females from low-income families have a more negative attitude toward entrepreneurship. This is because they must bear more family responsibilities and financial burdens and face more significant risks and pressures [52,81].Therefore, the following hypothesis is proposed:

**H9c:** Household income moderates the impact of ATE on EI.

Does vigorous implementation of EE in vocational colleges have the desired effect of enhancing EI? There have been numerous recent empirical studies on this topic, each of which has drawn different conclusions [82,83]. While some studies have found that EE significantly increases EI, others have found no such correlation between EE and EI [40]. From a perceptual perspective, the effect of household income on EI is evident [84]. Thus, this research investigates how household income influences the connection between EE and EI. The following hypothesis is proposed:

**H9d:** Household income moderates the impact of EE on EI.

**Fig 1:**
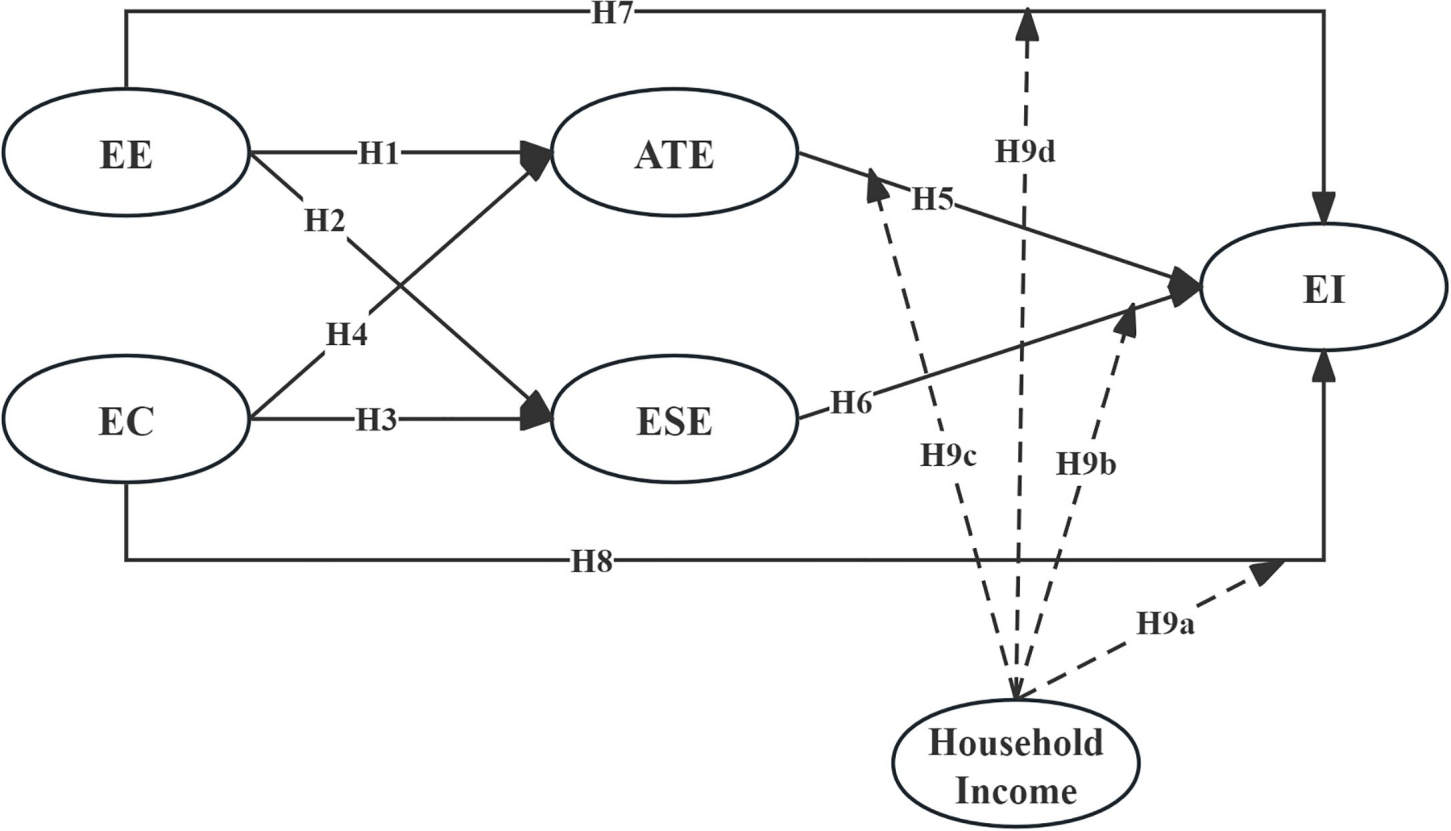
Theoretical framework.

Based on the hypotheses mentioned above, Fig 1 represents the theoretical model of this study.

In Fig 1, the influencing factors of EI include EE, EC, ATE, and ESE. Additionally, household income moderates the relationships between these four constructs and EI.

## 3. Methodology

### 3.1 Sample and data collection

This study aims to evaluate the relationship model of the proposed research hypothesis by gathering relevant data using the online questionnaire platform—Questionnaire Star (www.Sojump.com). The data collection period was from February 2023 to March 2023. Before administering the questionnaire survey, we ensured that every participant knew our research goals and obtained their written consent. All the people who participated in this research did so voluntarily, and their anonymity was explicitly promised. According to the research background, the sample participating in the survey comprised students attending vocational colleges. Therefore, to ensure the sample’s representativeness, we randomly selected students from vocational schools in Guangdong Province, Zhejiang Province, and Jiangxi Province. Each interviewee was currently enrolled in a vocational college, and they were conversant with their family and the entrepreneurship education provided by the college, so the given income range was used as the measurement method for annual household income in this study. Chi-square tests for household income (household_income_less_than_9000 = 41.16%, household_income_more_than_9000 = 53.21%; p = 0.512) were used to assess the sample’s representativeness. No statistically significant difference between the sample and population distribution was found, which indicated that the sample was highly representative.

In this study, we collected a total of 2149 valid questionnaires. The specific data on demographic characteristics are shown in Table 1. Most of the subjects in this study are female vocational college students between the ages of 18–20, with a percentage of around 69.71%. Referring to the table, most students have participated in EE with a ratio of 72.5%, while only a few students have not participated in EE. Lastly, according to the 2024 report on the income and expenditure of residents in China, the average annual family income for Chinese residents in 2023 was set at a threshold of 63,000 RMB (approximately 9,000 USD). Families with an average annual income exceeding 9,000 USD are classified as high-income, while those below this threshold are considered low-income. Therefore, this study has adopted this standard. Among the respondents, 1270 people have an average annual household income of less than 9000$, accounting for 59.52%.

**Table 1:** Profile and characteristics of females.

Characteristics	Item	Frequency	Percentage
**Age**	<18	367	17.08%
	18–20	1498	69.71%
	>20	284	13.22%
**Grade**	Freshman	1197	55.70%
	Sophomore	654	30.43%
	Junior	298	13.90%
**EE**	Accepted	1558	72.50%
	Unaccepted	491	22.85%
**Average annual household income**	less than 9000$	1270	59.52%
	More than 9000$	879	40.90%

### 3.2 Instrument development

The questionnaire is divided into two main parts: firstly, it collects demographic information from participants, followed by gathering self-reported responses to various constructs. This study utilizes validated scales to assess these constructs and has made necessary adjustments to the scales based on the research context and objectives. In addition to basic demographic information, the questionnaire also includes sections on EE, EC, ATE, ESE, and EI. Each item is rated using a Likert 5-point scale, ranging from 1 for "Strongly Disagree" to 5 for "Strongly Agree." A higher Cronbach’s alpha indicates greater internal consistency of the scale. The construction process of the questionnaire and its reliability and validity analyses are described as follows.

1. EE Scale
  The EE Scale is adapted from the study by Westhead & Solesvik and includes 8 items (for example, "I actively take courses on entrepreneurship education.") [85]. The Cronbach’s alpha for this scale is 0.899, indicating good internal consistency.
2. EC Scale
  The EC Scale is adapted from the research by Zhao et al. and includes 5 items (for example, "I can adapt to the new external environment and establish good relationships or partnerships with others.") [82]. The Cronbach’s alpha for this scale is 0.917, demonstrating a high level of reliability.
3. ATE Scale
  The ATE Scale draws from the study by Liu and includes 5 items (for example, "As long as I have the opportunity and resources, I am willing to start a business.") [37]. The Cronbach’s alpha value for this scale is 0.862, showing good reliability.
4. ESE Scale
  The ESE Scale is adapted from the research by Zhao et al. and contains 4 items (for example, "I understand the necessary details of starting a company.") [86]. The Cronbach’s alpha value for this scale is 0.858, indicating a high level of reliability.
5. EI Scale
  The EI Scale is adapted based on the research by Gelderen and includes 4 items (for example, "I once considered running my own company.") [87]. The Cronbach’s alpha value for this scale is 0.862, indicating good reliability.

### 3.3 Data analysis

The data is analyzed using a partial least squares structural equation model (PLS-SEM) [88]. Smart PLS 4.0 software is used for the model analysis. There are several reasons why PLS is chosen as the analysis tool in this study: (1) allows prediction models grounded in empirical data; (2) estimates complicated path models involving latent variables [89]. (3) multi-item observations; (4) the data is not requested to be distributed normally. This software has been regarded as a panacea in management science and behavioral research [90]. Smart PLS is applied to perform two steps: measurement model and structural model.

## 4. Results

### 4.1 The outer model

The measurement model is evaluated before conducting PLS-SEM estimation for hypotheses testing to ensure that the constructs are correctly significantly related to the proposed variance. Consequently, to assess the measurement model, the validity and reliability of the multi-item measures are examined.

An evaluation of the individual item reliability is conducted by scrutinizing the factor loadings of each variable. According to Byrne, a factor loading score of over 0.60 exhibits a high level of reliability [91]. It has been determined that each item showcased factor loading above 0.60 in Table 2.

**Table 2:** Outer model.

Constructs	Factor loadings	α	CR	AVE
**ATE**	0.807	0.862	0.866	0.645
	0.832			
	0.777			
	0.758			
	0.839			
**EC**	0.822	0.917	0.920	0.750
	0.852			
	0.877			
	0.897			
	0.879			
**EE**	0.757	0.899	0.905	0.585
	0.788			
	0.725			
	0.720			
	0.690			
	0.808			
	0.831			
	0.788			
**EI**	0.835	0.862	0.865	0.708
	0.877			
	0.852			
	0.801			
**ESE**	0.758	0.858	0.884	0.702
	0.792			
	0.911			
	0.882			

The composite reliability (CR) and Cronbach’s Alpha (α) evaluate the internal consistency. 0.7 for Cronbach’s Alpha is regarded as acceptable. In this study, Cronbach’s Alpha ranges from 0.862 to 0.917, meeting the criteria. In addition, according to Hair et al., CR values between 0.60 and 0.70 are acceptable, 0.70 and 0.90 are generally considered satisfactory, and values above 0.90 (and especially> 0.95) are not desirable [92]. The CR for all items in this study, which varied from 0.865 to 0.920, complied with the abovementioned criteria.

Convergent validity, determined using the Average Variance Extracted (AVE), is the variance between two or more items that concur when measuring similar constructs. Following the widely accepted threshold value of 0.5, this study’s latent variable AVE values are between 0.645 and 0.750, which are acceptable for the entire model [93]. This demonstrates that the variables meet the conditions for convergent validity.

Discriminant validity refers to the degree to which one construct can be genuinely distinguished from another (Zaiţ & Bertea, 2011). One method for differentiating validity tests is to apply the Fornell-Larcker criterion. It compares the AVE values’ square root to the latent variable’s correlations [93]. Per this criterion, the off-diagonal components in the relevant rows and columns are smaller than the diagonal values in bold, indicating adequate discriminant validity. Table 3 displays that the square root of the AVE of each construct is greater than its highest correlation with any other construct. Consequently, the results fulfill the requirements.

**Table 3:** Fornell-Larcker criterion.

Variables	ATE	EC	EE	EI	ESE
ATE	0.803				
EC	0.560	0.866			
EE	0.613	0.735	0.765		
EI	0.621	0.695	0.608	0.842	
ESE	0.519	0.492	0.485	0.539	0.838

The Heterotrait-Monotrait (HTMT), put forth by Henseler et al. , is the second criterion to evaluate discriminant validity [93]. All HTMT values, according to Gold et al. , are under 0.90 [94]. Table 4 depicts the discriminant validity of the measurement model using the HTMT criteria. All values fall below the critical threshold of 0.90. Consequently, the results fulfill the requirements.

**Table 4:** HTMT.

**Variables**	**ATE**	**EC**	**EE**	**EI**	**ESE**
**ATE**					
**EC**	0.627				
**EE**	0.679	0.808			
**EI**	0.716	0.778	0.676		
**ESE**	0.586	0.545	0.535	0.616	

### 4.2 The inner model

The structural model (inner model) analysis is carried out to guarantee that the structural model constructed for this study is accurate and robust. Several indicators can be used to analyze the inner model in this study, including the multi-collinearity test, significance test through the estimation of the path coefficients, coefficient of determination (R^2^), and effect size (f^2^).

#### 4.2.1 Collinearity test

The collinearity test is carried out to identify the issue of multi-collinearity in the model structure [92]. Following this rule of thumb, all the VIFs are lower than 3.3 [92]. The calculation collinearity test in Table 5 reveals that the VIF score ranges from 1.822 to 2.519, indicating no multi-collinearity problems.

**Table 5:** Collinearity statistics of the inner model.

**Variables**	**ATE**	**EC**	**EE**	**EI**	**ESE**
**ATE**				1.822	
**EC**	2.173			2.339	2.173
**EE**	2.173			2.519	2.173
**EI**					
**ESE**				1.504	

#### 4.2.2 Path hypothesis

In the PLS-SEM model, the goal of the significance test is to determine how exogenous variables affect endogenous variables. The summary of the results is presented in Table 6. The EE has a significantly positive effect on ATE (β = 0.438; t = 14.681; p = 0.000) and ESE (β = 0.270; t = 8.550; p = 0.000). Therefore, H1 and H2 are approved. The data show a significantly positive correlation between EC and ESE (β = 0.294; t = 9.720; p = 0.000). So, H3 is approved as well. Additionally, since a significantly positive correlation is found between the EC and ATE of female students, H4 is validated (β = 0.238; t = 7.631; p = 0.00). There is statistical significance between ATE and ESE on EI. Hence, H5 (β = 0.263; t = 10.270; p = 0.00) and H6 (β = 0.168; t = 8.731; p = 0.000) is approved as well. There appears to be no significant relationship between EE and EI (β = 0.052; t = 1.746; p = 0.081). Hence, H6 is not supported. As for the eighth hypothesis, it is proposed that EC has a direct and statistically significant impact on EI. The data confirm this conclusion (β = 0.427; t = 13.889; p = 0.000).

**Table 6:** The results of path coefficients.

Relationship	Path Coefficient (β)	t-value	P-value	Results
**EE→ATE**	0.438	14.681	0.000***	Supported
**EE→ESE**	0.270	8.550	0.000***	Supported
**EC→ESE**	0.294	9.720	0.000***	Supported
**EC→ATE**	0.238	7.631	0.000***	Supported
**ATE→EI**	0.263	10.270	0.000***	Supported
**ESE→EI**	0.168	8.371	0.000***	Supported
**EE→EI**	0.052	1.746	0.081	Not supported
**EC→EI**	0.427	13.889	0.000***	Supported

#### 4.2.3 Coefficient of Determination (R^2^)

The coefficient of determination (R^2^) value indicates how much the independent variable predicts the latent dependent variable. Meanwhile, the R^2^ test is to perform the robustness of corrections from predictions with the criterion of 0.67 (robust), 0.33 (moderate), and 0.19 (weak) [95]. EI has a moderate coefficient of determination (R^2^ = 0.582) (Table 7), indicating that this endogenous latent construct is explained by up to 58.2%.

**Table 7:** The predictive power of construct.

Variables	R square
ATE	0.402
EC	
EE	
EI	0.582
ESE	0.275

#### 4.2.4 Effect size (f^2^)

The size effect test (f^2^) seeks to determine the magnitude of the external latent variable’s influence on the structural model [92]. For f^2^, the size effect test (f^2^) is determined by several criteria: small (0.02), medium (0.15), and large (0.35) [96]. The effect size (f2) values are acceptable for the two exogenous latent variables, EC and EE, which are 0.186 and 0.030, respectively. Additionally, the f ^2^ index of the ATE and ESE is small (f^2^ <0.15) (Table 8).

**Table 8:** The effect size.

	**ATE**	**EC**	**EE**	**EI**	**ESE**
**ATE**				0.091	
**EC**	0.044			0.186	0.055
**EE**	0.147			0.030	0.046
**EI ESE**				0.045	

### 4.3 The Multi-group analysis for the Moderator Effects

A multi-group analysis is employed to examine the moderating effect of household income. This method, which is a non-parametric significance test based on the bootstrapping results, has been utilized to compare PLS estimates in different groups [97].

Before performing multi-group analysis, groups must be generated in the data. Based on the background of this study, we divide the data into high-household-income groups and low-household-income groups [98]. According to Leguina, to meet the minimum R^2^ of 0.10 at a 5% significance level, both subgroups must exceed to137 [99], as the maximum number of arrows pointing towards EI in this study is 4. Meanwhile, Hair et al. point out that while the sizes of two subpopulations need not be identical, they must be comparable [100]. The guideline for considering group sample size differences is that one group is less than 50% of the other. Therefore, both sample groups in this study meet the requirements.

However, it is required to test for measurement invariance to acquire reliable results [101]. The MICOM method proposed by Henseler et al. is used to validate measurement invariance [101]. Henseler et al. argue that configural invariance, compositional invariance, and equality of composite mean values and variances must be established to perform a multi-group analysis [101].

Firstly, the study guarantees uniformity between the two groups’ data processing, measurement and structural models, and algorithm settings (using the same questionnaires in both cases). Thus, it can be said that configural invariance has been established.

The second step of the MICOM procedure is designed to test compositional invariance. In step 2, Permutation tests are conducted. Constitutive invariance exists when the original correlation is equal to or larger than 5% of the quantile correlation (shown in the 5% column) [102]. As can be seen in Table 9, the MICOM second-stage findings report shows that the original correlation is always equal to or greater than the 5%-quantile. Thus, compositional invariance has been established for all the constructs.

**Table 9:** MICOM Step 2 results report.

	Original correlation	Correlation permutation means	5.00%	Permutation p-values
**ATE**	1.000	1.000	1.000	0.111
**EC**	1.000	1.000	1.000	0.061
**EE**	0.999	1.000	0.999	0.220
**EI**	1.000	1.000	1.000	0.065
**ESE**	1.000	1.000	1.000	0.270

The third step is to evaluate the composite equality of mean values and variances across groups. Two conditions must be satisfied. Firstly, the first column (mean original difference) must be a number within the 95% confidence interval. Secondly, the data in column one (variance original difference) must be a number that falls within the 95% confidence interval. Full invariance is constructed if both conditions are satisfied. Additionally, partial invariance is established when a construct passes only one of the two confidence interval tests. According to Tables 10 and 11, partial invariance has been established.

**Table 10:** MICOM Step 3 results report—Part 1.

	Mean original difference (males-females)	Mean permutation mean difference (males-females)	2.50%	97.50%	Permutation p-values
**ATE**	0.014	0.000	-0.096	0.083	0.210
**EC**	0.043	0.001	-0.090	0.086	0.330
**EE**	0.012	0.001	-0.088	0.086	0.061
**EI**	0.047	-0.001	-0.087	0.077	0.302
**ESE**	0.028	-0.001	-0.091	0.085	0.040

**Table 11:** MICOM Step 3 results report—Part 2.

	Variance original difference (high-low)	Variance permutation mean difference (high-low)	2.50%	97.50%	Permutation p-values
**ATE**	-0.457	0.003	-0.152	0.167	0.000
**EC**	-0.620	0.000	-0.157	0.156	0.000
**EE**	-0.649	0.000	-0.165	0.159	0.000
**EI**	-0.383	0.001	-0.152	0.143	0.000
**ESE**	-0.313	0.002	-0.108	0.121	0.000

After the partial invariance is established, it is necessary to determine whether the path coefficients of the two groups are significantly different. We will first analyze each group separately to see if there are variations between the groups. The analysis results are shown in Table 12. The following step is to ascertain whether or not the difference between the two groups is statistically significant. The results of the permutation test can be used for this purpose. A permutation p-value of less than or equal to 0.10 indicates a significant difference between the two groups. Table 13 displays the Bootstrapping results of the multi-group analysis.

**Table 12:** Bootstrapping results for high and low-household-income families separately.

Path	Original (high)	Mean (high)	STDEV (high)	t value (high)	p-value (high)	Original (low)	Mean (low)	STDEV (low)	t value (low)	p-value (low)
**ATE -> EI**	0.351	0.351	0.033	10.584	0.000***	0.218	0.219	0.035	6.288	0.000***
**EC -> EI**	0.266	0.266	0.037	7.207	0.000***	0.525	0.524	0.043	12.189	0.000***
**EE -> EI**	0.123	0.124	0.034	3.618	0.000***	0.007	0.008	0.042	0.174	0.861
**ESE -> EI**	0.170	0.171	0.030	5.660	0.000***	0.162	0.162	0.024	6.695	0.000***

**Table 13:** Multi-group analysis results.

Path	Path coefficients original (high)	Path coefficients original (low)	Path coefficients original difference (high- low)	Path coefficients permutation means difference (high- low)	2.50%	97.50%	Permutation p-values
**ATE -> EI**	0.351	0.218	0.133	0.000	-0.102	0.092	0.008
**EC -> EI**	0.410	0.619	-0.209	-0.001	-0.122	0.115	0.000
**EE -> EI**	0.298	0.156	0.142	0.001	-0.122	0.121	0.027
**ESE -> EI**	0.170	0.162	0.008	-0.001	-0.090	0.081	0.859

As shown in Tables 12 and 13, it can be determined that there is no notable disparity in the impact of ESE on EI among females categorized by high and low household income (p = 0.859). However, the relationship between ESE and EI is more pronounced in low household income than in high household income. In addition, household income has a significant moderating effect on the relationship between ATE and EI, EC and EI, and EE and EI.

### 4.4 Common method bias (CMB)

Common method bias (CMB) was assessed using two methods. First, the Harman’s single-factor test indicated that no single factor accounted for the majority of the variance [103]. The test produced a maximum single factor explaining 45.295% of the variance, which is well below the critical threshold of 50% [103]. Secondly, the marked variable method was employed, which involves introducing a theoretically unrelated marker variable into the research model to test for common method bias [104]. The highest shared variance estimate with other variables was 0.0311 (3.11%), which is significantly low [105]. Therefore, based on the results of these two tests, it can be inferred that there is no significant common method bias present.

## 5. Discussion

This research examined the factors affecting the EI of female students in vocational colleges. It was posited that the predictive factors for EI include several independent variables, specifically EE, EC, ATE, and ESE. Meanwhile, this study analyzed how household income moderates the relationship between EE, ESE, ATE, EC, and EI. Moreover, the research model underwent testing utilizing the structural equation modeling technique, yielding results that indicated a good fit with the gathered data. Most of the hypothesized relationships between constructs have been substantiated, accounting for 58.2% of the total variance in the EI. In the subsequent paragraphs, our findings are discussed concerning the research questions raised in the introduction.

The result discovers a significant relationship between EE and ATE. The outcome is consistent with the findings provided by Maheshwari [20]. Maheshwari mention that reasonable and effective EE can cultivate students to form a positive ATE [20]. In this study, on the one hand, female students can learn theoretical knowledge, practical experience, and case analysis related to entrepreneurship through EE. This knowledge enables female students to understand the entrepreneurial process better, including risks, challenges, and opportunities. An accurate and objective understanding of entrepreneurship can help female college students form a positive ATE. On the other hand, EE can cultivate competence in innovation, marketing, business model design, financial management, etc. This can also strengthen the ATE of female vocational college students.

There is a significant positive relationship between EE and ESE. In this research, firstly, EE involves knowledge and skills related to starting and managing a business. This knowledge can boost female students’ self-confidence in entrepreneurship. Secondly, EE exposes female students to successful female entrepreneurs. By learning about entrepreneurial experiences and lessons of successful female entrepreneurs, female students may have more confidence in becoming successful entrepreneurs. Finally, EE can provide support and encouragement from mentors and peers, contributing to female students’ confidence in their EC. In summary, EE can enhance the ESE of female students in vocational colleges. The findings align with prior literature [61,106].

According to the findings of the research hypothesis test, EC has a significant impact on ESE. The findings of this study confirm the research results of Reis [52], who discovered that a higher ESE would follow a higher EC. In the context of this study, on the one hand, female students with high EC may have already acquired the knowledge and skills necessary for successful business creation and operation. This knowledge and skills can strengthen their belief in their competence to succeed in entrepreneurship. On the other hand, female students inevitably face setbacks and failures in the entrepreneurial process. If they have EC such as analysis, criticism, and dialectical thinking, they can analyze and summarize their entrepreneurial experiences and lessons. In that case, they are more likely to enhance their ESE.

The EC has a positive effect on ATE. Female students can learn real-world entrepreneurship cases during their studies at vocational colleges, gain practical experience, and gain the professional skills required for real entrepreneurship, such as marketing and financial management. This competence can help female students in vocational college develop a positive ATE. The results are also consistent with the research by Li [16], who found that EC obtained by students influenced the attitude of students toward entrepreneurship.

There is a significant positive correlation between ATE and EI. Female students in vocational colleges focus on practical skill development and are closer to the real market and industry demands. This makes them more likely to discover innovation points in entrepreneurial opportunities and business models, which helps them develop a more positive ATE and enhances their EI. In addition, female students in vocational colleges with a more positive ATE may be more confident and able to face challenges and difficulties bravely, resulting in a higher EI. This finding agrees with the results of prior studies [66].

The findings point to a direct relationship between ESE and EI. On the one hand, vocational colleges emphasize cultivating students’ practical skills and providing them with more hands-on experience opportunities. The accumulation of these experiences and skills can effectively help female students improve their ESE. Female students with higher ESE tend to believe they can start and manage a business. This confidence can be transformed into a higher level of EI. On the other hand, female students in vocational colleges may have opportunities to establish connections with other students, faculty members, and industry professionals and receive their guidance and support. These valuable resources can further enhance female students’ ESE and EI. Thus, the findings lend credence to the opinion that a full sense of ESE can improve college students’ confidence in their EC and further stimulate their EI [9].

Unexpectedly, the findings of this research suggest that there is no significant correlation between EE and EI. This study’s results differ from previous scholars who have examined similar issues in the context of European countries [58,107–109]. Firstly, some female students may be more inclined to choose traditional career paths than entrepreneurship, even if vocational schools provide good EE. Secondly, if the quality of EE in vocational schools is low, it may not offer sufficient inspiration and support to promote female students’ EI. In this case, even if female students receive EE, they may be unable to increase their EI effectively. Finally, external factors such as economic, social, and cultural factors influence EI. If the external environment is not conducive to entrepreneurship or entrepreneurs face too many risks and challenges, it may inhibit female students’ EI and render EE ineffective.

Consistent with the findings of Turulja [53], the current study discovers that EC is one of the significant predictors of EI. In the context of this study, on the one hand, when female students have EC, such as market research, business model design, and team management, they will have more confidence and motivation to pursue entrepreneurial goals. This confidence and motivation can promote an increase in EI. On the other hand, EC is the foundation of entrepreneurial success. It can help female students better grasp business opportunities, resolve and avoid risks, and continue to learn and improve during the entrepreneurial process. Improving this competence can increase the success rate of female students’ entrepreneurship, thereby increasing their EI. In addition, female students with EC can better understand the market and consumer needs, design products and services that meet market demand, and manage and operate enterprises better, all of which can improve the feasibility of entrepreneurship.

H9a is supported. This means a significant difference in the relationship between EC and EI among female students based on household income levels. Entrepreneurship allows low-income female students to escape poverty and improve their living conditions. However, starting a business requires entrepreneurial skills and resources, which are often lacking in low-income households. To overcome these challenges, low-income female students must enhance their EC. Therefore, the intention of low-income female college students to start a business may be more influenced by their EC. In other words, the higher their EC, the more feasible they consider entrepreneurship viable. On the other hand, high-income female students have more financial resources, more robust financial security, and a wider range of career choices. They are more likely to focus on entrepreneurship’s desirability than their EC. Therefore, compared to low-income families, the impact of EC on the EI of high-income female students is smaller. The conclusion aligns with Wang & Jiang [110].

Based on the findings of the study, H9b is rejected. On the one hand, ESE mainly comes from personal experiences, skills, and self-evaluation rather than family income level. Therefore, female college students from different household income backgrounds show no differences in ESE. On the other hand, in addition to household income, many other factors (such as personal education level, work experience, personality traits, etc.) may have a stronger impact on the relationship between ESE and EI, thereby masking the influence of household income level on this relationship. The findings of this study are consistent with previous studies [111].

Household income significantly moderates the relationship between ATE and EI. Hence, H9c is confirmed. In other words, significant differences exist in the relationship between ATE and EI among household income levels. Higher-income families have more social resources and can provide better entrepreneurial support. Therefore, female college students from higher-income families have a more positive ATE and a greater impact on EI. Lower-income families are more concerned with livelihood, basic economic needs, and stable employment opportunities and may be more cautious about entrepreneurial risks and uncertainties. Therefore, the ATE of female students from lower-income families may be relatively weak, and their impact on EI may be more limited. This outcome supports Cui’s conclusion [107].

The findings obtained through the multi-group analysis confirm that the relationship between EE and EI among female college students from high household incomes is stronger than those from low-income families. On the one hand, female students from higher-income families may receive high-quality EE and have access to more entrepreneurial knowledge and skills. Female students from lower-income families are less likely to receive high-quality EE. On the other hand, female students from higher-income families may have easier access to successful entrepreneurs and have more opportunities to try entrepreneurship. In contrast, female students from lower-income families may lack such opportunities. The result of H9d is congruent with previous studies [81].

## 6. Implications

### 6.1 Theoretical implications

The first theoretical contribution of this study is the expansion of the application of the TPB. Based on a literature review and the TPB, an assessment model of EI for female students in vocational college was developed. This model considers internal factors such as ATE, EC, and ESE and external factors such as EE and household income.

This study empirically validates the factors influencing the EI of female students in vocational college, indicating that all relationships except for the direct effect between EE and EI have been confirmed. That is to say, ATE, ESE, and EI can directly or indirectly enhance the EI of female students in vocational colleges. This is the second theoretical contribution of this study.

This study also analyzes the moderating effect of household income on the relationship between each construct and EI. Previous studies only discussed the relationship between the constructs. To our knowledge, this study is the first to test the moderating effect of household income in the assessment model of EI for female students in a vocational college. This is the third theoretical contribution of this study.

The fourth theoretical contribution of this study involves the explanatory power of the model. The model has an explanatory power of 58.2% for the variance of EI of female students in vocational college, which is relatively strong and reaches a medium to a high level. Compared with previous models, it has been greatly improved.

### 6.2 Practical implications

This study’s findings are of significant importance for vocational education and entrepreneurship development in China. It highlights how attributes such as entrepreneurial attitude and self-efficacy substantially influence the entrepreneurial intentions of female students in Chinese vocational colleges. These insights can guide educational policymakers and curriculum designers in tailoring programs that enhance these attributes among students.

Firstly, vocational colleges in China should integrate comprehensive entrepreneurship education that is culturally and contextually relevant, aiding female students not only in acquiring essential entrepreneurial skills but also in developing a robust entrepreneurial mindset that aligns with the societal and economic frameworks of China.

Secondly, the government can utilize these insights to formulate policies that more robustly support female entrepreneurship. These might include financial incentives, tax breaks, and targeted entrepreneurial guidance. Additionally, considering the role of household income highlighted by the research, policies could provide greater support to students from low-income families to bridge gaps and promote a more equitable entrepreneurial environment.

Furthermore, the study indicates that family support is crucial in fostering entrepreneurial intentions. In the Chinese context, where family influence is prominent, encouraging families to support the entrepreneurial aspirations of female members could be key. This can be facilitated through community-based programs and national campaigns that educate and engage families in the entrepreneurial process.

Lastly, the results suggest the need for localized support systems that consider the unique challenges faced by female entrepreneurs in China. These include addressing cultural stereotypes, providing networking opportunities, and securing access to markets crucial for the sustainability of local enterprises.

By addressing these practical implications, vocational colleges, policymakers, and China’s entrepreneurial ecosystem can better support and empower female entrepreneurs, contributing to the country’s broader economic development goals.

## 7. Limitations

The present study has a few limitations that must be taken into account. Firstly, this study’s generalizability is limited as it only selected samples from three provinces in China, namely Guangdong, Guangxi, and Jiangxi provinces. Future researchers should validate the conclusions by performing further research in other circumstances, given that entrepreneurial activity differs by country and economic level. Secondly, the cross-sectional data of the questionnaire used in this study only represents the situation at a specific time and may not accurately reflect the dynamic development trend. Future research could adopt mixed methods, such as longitudinal or experimental design, to better understand the variables’ dynamic relationships. Lastly, future research could consider other moderator variables, including but not limited to entrepreneurial resilience and risk perception, to enhance a comprehensive analysis of the factors influencing the EI of female students in vocational colleges.

## 8. Conclusion

This study aims to explore the factors influencing the EI of female college students in Chinese vocational colleges based on the Theory of Planned Behavior, as well as whether household income moderates the relationships between EE, ATE, EC, ESE, and EI. A literature review indicates that EE, ATE, EC, and ESE are significant predictors of EI among female college students in Chinese vocational colleges. This study collected 2,149 valid questionnaires through an online survey platform. The impact of EE, ATE, EC, and ESE on EI was tested using structural equation modeling. The results show that all hypotheses were supported except for the non-significant relationship between EE and EI. Additionally, household income did not significantly moderate the relationship between ESE and EI, but it did significantly moderate the relationships between ATE, EC, EE, and EI. Theoretically, this study not only expands the application of the Theory of Planned Behavior but also considers the internal and external factors affecting the EI of female students in vocational colleges. Furthermore, the study analyzes the moderating role of household income in the relationships between various constructs and EI. Practically, the study suggests that vocational colleges should help female students acquire entrepreneurial knowledge and resources through various means, and governments should support female student entrepreneurship through initiatives such as entrepreneurial funding, tax incentives, and entrepreneurial guidance.

## Supporting information

S1 Raw data (SAV)

S1 Appendix (DOCX)
