# Supplementary material for: Factors influencing female students’ entrepreneurial intention in vocational colleges: A multi-group analysis based on household income
Source: PLoS One. 2024 May 23;19(5):e0304232. doi: 10.1371/journal.pone.0304232 (PMC11115265; doi:10.1371/journal.pone.0304232)
Supplement: S1 Appendix — (DOCX) [file pone.0304232.s002.docx]

Appendix A. Instruments

| Construct | Item | Source |
| --- | --- | --- |
| ESE | ESE1: It’s not difficult for me to start and run a company.  ESE2: I understand the necessary details of starting a company.  ESE3: I have the confidence to start a business successfully.  ESE4: There is an excellent possibility of success in my own business. | ([Zhao et al., 2005](#_ENREF_140)) |
| ATE | ATE1: As long as I have the opportunity and resources, I am willing to start a business. | ([Liu et al., 2019](#_ENREF_78)) |
|  | ATE2: Entrepreneurship will bring me greater satisfaction. |  |
|  | ATE3: The advantages of starting a business outweigh the disadvantages.  ATE4: For me, choosing to start a business can fully realize my self-worth.  ATE5: I believe entrepreneurship can bring me greater value than other options. |  |
| EE | EE1: I actively take courses on entrepreneurship education EE2: I accept the initiative to attend entrepreneurship lectures or reports  EE3: I have taken the initiative to participate in entrepreneurial skills training or simulation exercises organized by the school  EE4: I have actively participated in entrepreneurial activities organized by learning organizations.  EE5: During my study, my vocational college attached importance to entrepreneurship education.  EE6: The practical projects of entrepreneurship education organized by higher vocational schools have greatly helped me  EE7: During my study, the entrepreneurship education courses provided by teachers in higher vocational colleges were very enlightening to me  EE8: The courses in entrepreneurship education in vocational colleges have improved my entrepreneurial knowledge reserve. | ([Westhead & Solesvik, 2016](#_ENREF_128)) |
|  |  |  |
| EC | EC1: I can adapt to the new external environment and establish good relationships or partnerships with others  establish good relationships or partnerships with others | (Zhao et al., 2023) |
|  | EC2: I can efficiently recruit like-minded partners with similar value orientations |  |
|  | EC3: I can use theoretical knowledge to generate new ideas and discoveries in different fields  EC4: I can effectively utilize various resources for related entrepreneurial activities  EC5: I can effectively explore potential market opportunities and make objective assessments. |  |
| EI | EI1: I once considered running my own company.  EI2: I will choose to start a business in the next 3 or 5 years.  EI3: If I have the opportunity and I am free to make decisions, I will choose to start my own business.  EI4: Considering my current actual situation and various limitations (such as lack of funds), I will still choose to start my own business | ([Gelderen, 2006](#_ENREF_50)) |
